# *Lactobacillus sakei* WIKIM30 Ameliorates Atopic Dermatitis-Like Skin Lesions by Inducing Regulatory T Cells and Altering Gut Microbiota Structure in Mice

**DOI:** 10.3389/fimmu.2018.01905

**Published:** 2018-08-14

**Authors:** Min-Sung Kwon, Seul Ki Lim, Ja-Young Jang, Jieun Lee, Hyo Kyeong Park, Namhee Kim, Misun Yun, Mi-Young Shin, Hee Eun Jo, Young Joon Oh, Seong Woon Roh, Hak-Jong Choi

**Affiliations:** ^1^Microbiology and Functionality Research Group, World Institute of Kimchi, Gwangju, South Korea; ^2^Division of Animal Science, Chonnam National University, Gwangju, South Korea

**Keywords:** atopic dermatitis, lactic acid bacteria, *Lactobacillus sakei*, regulatory T cells, gut microbiota, kimchi

## Abstract

*Lactobacillus sakei* WIKIM30 is a Gram-positive facultative anaerobic bacterium isolated from kimchi, a Korean fermented vegetable food. In this study, we found that WIKIM30 promoted regulatory T cell (Treg) differentiation by inducing dendritic cells with tolerogenic properties. The production of the T helper (Th) 2-associated cytokine interleukin (IL)-4 was decreased, but that of the Treg-associated cytokine IL-10 was increased in splenocytes from ovalbumin-sensitized mice treated with WIKIM30. We also investigated the inhibitory capacity of WIKIM30 on the development of 2,4-dinitrochlorobenzene-induced atopic dermatitis (AD), a Th2-dominant allergic disease in mice. Oral administration of *L. sakei* WIKIM30 significantly reduced AD-like skin lesions and serum immunoglobulin E and IL-4 levels while decreasing the number of CD4^+^ T cells and B cells and the levels of Th2 cytokines (IL-4, IL-5, and IL-13) in peripheral lymph nodes and enhancing Treg differentiation and IL-10 secretion in mesenteric lymph nodes. In addition, WIKIM30 modulated gut microbiome profiles that were altered in AD mice, which showed increases in *Arthromitus* and *Ralstonia* and a decrease in *Ruminococcus* abundance. These changes were reversed by WIKIM30 treatment. Notably, the increase in *Ruminococcus* was highly correlated with Treg-related responses and may contribute to the alleviation of AD responses. Together, these results suggest that oral administration of *L. sakei* WIKIM30 modulates allergic Th2 responses enhancing Treg generation and increases the relative abundance of intestinal bacteria that are positively related to Treg generation, and therefore has therapeutic potential for the treatment of AD.

## Introduction

Atopic dermatitis (AD) is an allergic skin disease accompanied by chronic inflammation that is characterized by severe itching, redness, dryness, and eczematous skin lesions and is associated with high serum level of immunoglobulin (Ig)E (1). Although the pathogenesis of AD is not fully understood, it is known to be caused by immune dysregulation resulting from the complex interaction of environmental and genetic factors. T helper (Th) 2 responses are predominant in the acute stage of AD, whereas Th1 and Th17 responses contribute to disease pathogenesis at the chronic stage (2–4). Thus, AD lesions show accumulation of infiltrated inflammatory cells and destruction of the skin barrier due to an imbalance in Th responses (2).

Various therapeutic approaches have been used to alleviate chronic inflammation in AD, including the administration of probiotics in recent therapeutic trials (5, 6). Probiotics are live nonpathogenic bacteria that provide health benefits when consumed such as strengthening of the gastrointestinal (GI) epithelial barrier, inhibiting the growth of enteric pathogens, improving the balance of gut microbiota, and enhancing host intestinal immune function (7, 8). Immune homeostasis in the gut is achieved through interaction with epithelial cells, dendritic cells (DCs), macrophages, and lymphocytes. DCs are specialized antigen-presenting cells that stimulate immune responses by modulating T cell function (9, 10). Ingested probiotics induce DC maturation and migration to the mesenteric lymph node (MLN), where they increase immune tolerance by stimulating the generation of regulatory T cells (Tregs) (11). CD103^+^ DCs are critical for mediating immune tolerance in the GI system (12, 13). During the allergic response, Tregs migrate into peripheral tissues and inflammation draining lymph nodes, leading to Treg-mediated immune suppression of Th2 immune responses (14–17).

Microorganisms colonizing the gut play important roles in host health, including digestion of dietary components, production of metabolites (fatty acids, glycolipids, and vitamins), and regulation of immune system maturation (18). Maintaining proper gut microbiota is critical for host immune homeostasis; perturbation of the gut microbiota balance is correlated with the development and progression of various disorders including allergic diseases (19, 20). Modulating gut microbiota composition and function is a promising strategy for their treatment. Emerging clinical evidence indicates that probiotics can restore gut microbiota composition and promote the beneficial functions of gut microbiota, thereby improving disease symptoms (21). However, the underlying mechanisms on how this alleviates diseases remain to be elucidated.

In this study, we isolated *Lactobacillus sakei* strain WIKIM30 from kimchi, a Korean traditional fermented food, and investigated its immunomodulatory properties in a mouse model of AD induced by 2,4-dinitrochlorobenzene (DNCB). We found that WIKIM30 induced the transformation of DCs to a tolerogenic form that promoted Treg differentiation *in vitro* and improved AD symptoms *in vivo* through modulation of immune responses and gut microbiome composition.

## Materials and Methods

### Isolation and Preparation of *L. sakei* WIKIM30

*Lactobacillus sakei* WIKIM30 was isolated from homemade kimchi in Korea. The kimchi was homogenized in a stomacher, and the homogenate was passed through the filter bag and diluted before it was spread onto a de Man, Rogosa, and Sharpe (MRS; BD Biosciences, Franklin Lakes, NJ, USA) agar plate that was then incubated at 30°C for 2 days. The resultant lactic acid bacteria (LAB) colonies were isolated by sequential culturing and identified based on the 16S rRNA gene sequence. Sequence data were aligned and compared to those in the GenBank database. A phylogenetic analysis of the 16S rRNA gene sequence in the isolate revealed a 99.86% similarity to that of *L. sakei*; it was thus deposited in the Korean Federation of Culture Collection as *L. sakei* KFCC 11625P.

WIKIM30 was cultured overnight at 30°C in MRS broth. The culture was diluted 1:200 in fresh medium and cultured for a second night for maximal growth. The optical density at 600 nm (OD_600_) was measured, and the number of colony-forming units (CFU) was determined from standard growth curves. For all cultured bacterial strains, an OD_600_ value of 1 corresponded to 1 × 10^8^ CFU/ml, which was confirmed by plating serial dilutions on MRS agar plates. After overnight culture, bacteria were washed in fresh, sterile phosphate-buffered saline (PBS; pH 7.4) and immediately administered to the mice, which received either sterile PBS or 2 × 10^9^ CFU bacteria in 200 µl PBS by intragastric gavage every day.

### *In Vitro* Culture and Stimulation of Murine Bone Marrow-Derived DCs (BMDCs)

Bone marrow (BM) cells were isolated and cultured as previously described (22, 23). Femora and tibiae from 6-week-old male BALB/c mice were removed and stripped of muscles and tendons. The bones were rinsed in PBS and then crushed with a mortar to release BM cells. After washes with Roswell Park Memorial Institute (RPMI)-1640 medium, BM cells (2 × 10^6^) were seeded in Petri dishes in 10 ml complete RPMI-1640 supplemented with 10% (v/v) fetal bovine serum, 100 U/ml penicillin, 100 µg/ml streptomycin, and 50 µM β-mercaptoethanol in the presence of 20 ng/ml murine granulocyte-macrophage colony-stimulating factor (GM-CSF; Peprotech, Rocky Hill, NJ, USA). The cells were incubated for 10 days at 37°C. On day 3, the culture medium was supplemented with fresh complete RPMI-1640 containing 20 ng/ml murine GM-CSF, and on day 8, the medium was replaced with fresh complete RPMI-1640 containing 20 ng/ml murine GM-CSF. On day 10, immature DCs were collected and seeded in a 96-well plate at 5 × 10^5^ cells/well. The cells were either left unstimulated or were stimulated with *L. sakei* WIKIM30 (1:5 cell to bacteria ratio) or LPS (100 ng/ml) for 24 h at 37°C. The culture supernatant was then collected and TNF-α, interleukin (IL)-12p70, and IL-10 levels were evaluated by flow cytometry using a Cytometric Bead Array kit (BD Biosciences, San Jose, CA, USA). For phenotypic analysis, cells were stained for the DC marker CD11c and CD11b; activated DC markers CD40, CD69, CD80, CD86, and MHCII; tolerogenic DC markers inducible T-cell costimulator ligand (ICOS-L), programmed death ligand (PD-L)1, and CD103; or appropriate isotype controls (BD Biosciences) and analyzed by flow cytometry. Data were analyzed with FlowJo software (Tree Star Inc., Ashland, OR, USA).

### BMDC/T Cell Cocultures

The BMDC and T cell coculture system was adapted from a previous study (24). Briefly, immature BMDCs were stimulated with 100 ng/ml of LPS or WIKIM30 for 24 h at 37°C. Unstimulated or stimulated BMDCs (1 × 10^5^ cells/well) were washed and cocultured with naïve CD4^+^ T cells (3 × 10^5^ cells/well) purified from the spleen of BALB/c mice in the presence of soluble anti-CD3 monoclonal antibody (mAb) (2 µg/ml) for 4 days. Naïve CD4^+^ T cells were purified using the Naïve CD4^+^ T Cell Isolation kit (130-094-131; Miltenyi Biotec, San Diego, CA, USA).

### Animal Studies

Wild-type male BALB/c mice were purchased from OrientBio Co. (Gwangju, Korea). Two to three mice were housed in each ventilated cage at a temperature of 22 ± 2°C and relative humidity of 55 ± 5% under a 12:12-h light/dark cycle in a pathogen-free animal facility at the World Institute of Kimchi. The mice were fed standard chow and had free access to water. To investigate the beneficial effects of LAB on AD, AD-like lesions were induced with DNCB (Sigma-Aldrich, St. Louis, MO, USA) as previously described (6). Mice were randomized into the following four groups (*n* = 5 each): naïve (treatment-naïve mice fed vehicle), negative control (mice treated with DNCB and fed vehicle), positive control (mice treated with DNCB and fed ketotifen at 1 mg/kg), and WIKIM30 (mice treated with DNCB and fed *L. sakei* WIKIM30). The dorsal skin was shaved, and 200 µl of 1% DNCB in acetone/olive oil (3:1) was applied twice a week. After 3 weeks, 0.2% DNCB was applied to the dorsal skin once a week. WIKIM30 or PBS (200 µl) was administered to the mice by intragastric gavage once daily. The vehicle and LAB were administered for 42 days. The severity of dermatitis in the dorsal skin of DNCB-induced AD mice was evaluated based on five symptoms (erythema/hemorrhage, edema/excoriation, erosion, scarring/dryness, and lichenification), which were scored as 0 (none), 1 (mild), 2 (moderate), or 3 (severe). Dermatitis score was defined as the sum of individual scores. On day 43, the mice were sacrificed under CO_2_, and blood samples, dorsal skin, spleen, MLNs, and peripheral (P)LNs (axillary and inguinal LNs) were collected for further analysis.

### Measurement of Total Serum IgE and Cytokine Levels

Blood samples were collected from mice after sacrifice, and serum samples were obtained by centrifugation (3,000 × *g*, 10 min). Serum IgE and IL-4 levels were measured using the OptEIA Mouse Sets (BD Biosciences). Single-cell suspensions from spleen, PLN, and MLN samples were obtained by mechanical disruption in 0.5 ml complete RPMI-1640. The cells were seeded in a 96-well plate at 5 × 10^5^ cells/well and cocultured with anti-CD3/CD28 mAbs (1 µg) for 24 h at 37°C. IL-4, -5, -10, and -13 and IFN-γ concentrations in the supernatant were quantitated by flow cytometry using a Cytometric Bead Array kit.

### Histological Analysis

The dorsal skin of mice was removed, fixed in 10% phosphate-buffered formalin, embedded in paraffin, and cut into sections that were stained with hematoxylin and eosin (H&E) for evaluation of edema. Other sections were stained with Toluidine Blue for detection of mast cells.

### Flow Cytometry Analysis

Mesenteric lymph nodes isolated from each group of mice and CD4^+^ T cells cocultured with LPS- or WIKIM30-treated BMDCs were labeled with fluorescein isothiocyanate-conjugated anti-mouse CD4, allophycocyanin (APC)-conjugated anti-mouse CD25, peridinin chlorophyll-Cy5.5-conjugated anti-mouse T cell receptor β (BD Biosciences), or phycoerythrin (PE)-conjugated anti-mouse Forkhead box (Fox)p3 (eBioscience, San Diego, CA, USA) mAb. Flow cytometry was performed on a FACSCanto II system (BD Biosciences), and data were analyzed using FlowJo software (Tree Star Inc., Ashland, OR, USA).

### Intracellular Cytokine Staining

Isolated peripheral lymph node cells were cultured in the presence of 20 ng/ml phorbol 12-myristate 13-acetate (Sigma-Aldrich), 1 mM ionomycin (Sigma-Aldrich), and 10 µg/ml brefeldin A for 4 h. Following surface antigen staining, cells were treated with fixation and permeabilization solutions (eBioscience) according to the manufacturer’s instructions and then stained with APC-conjugated anti-IFN-γ (clone XMG1.2; BD Biosciences) and PE-conjugated anti-IL-4 (clone 11B11; BD Biosciences) mAbs, with isotype-matched antibodies used as controls.

### Gut Microbiota Analysis

Fresh fecal samples were collected from mice and immediately stored at −150°C until processing. Fecal DNA was isolated using the FastDNA Spin kit (MP Biomedicals, Santa Ana, CA, USA). PCR amplification was performed using barcoded primers targeting the V3 to V4 region of the bacterial 16S rRNA gene. Amplicons were sequenced using the 250-bp paired-end read strategy on the MiSeq sequencing system (Illumina, San Diego, CA, USA). Fast length adjustment of short reads was used to merge reads based on the Greengenes database. Output data were analyzed using BIOiPLUG software (Chunlab, Seoul, Korea).

### Statistical Analysis

Statistical analysis was performed using Prism v.6.0 software (GraphPad Inc., La Jolla, CA, USA), and results are presented as mean ± SE. Treatment effects were evaluated with the Student’s *t*-test; *p* < 0.05, *p* < 0.01, and *p* < 0.001 was considered statistically significant. To examine the relationship between gut microbiota abundance and various parameters related to AD and WIKIM30 in mouse groups, a principal component analysis was performed using XLSTAT (Addinsoft, Paris, France).

## Results

### WIKIM30 Modulates DC and T Cell Function *In Vitro*

**Figure 1:**
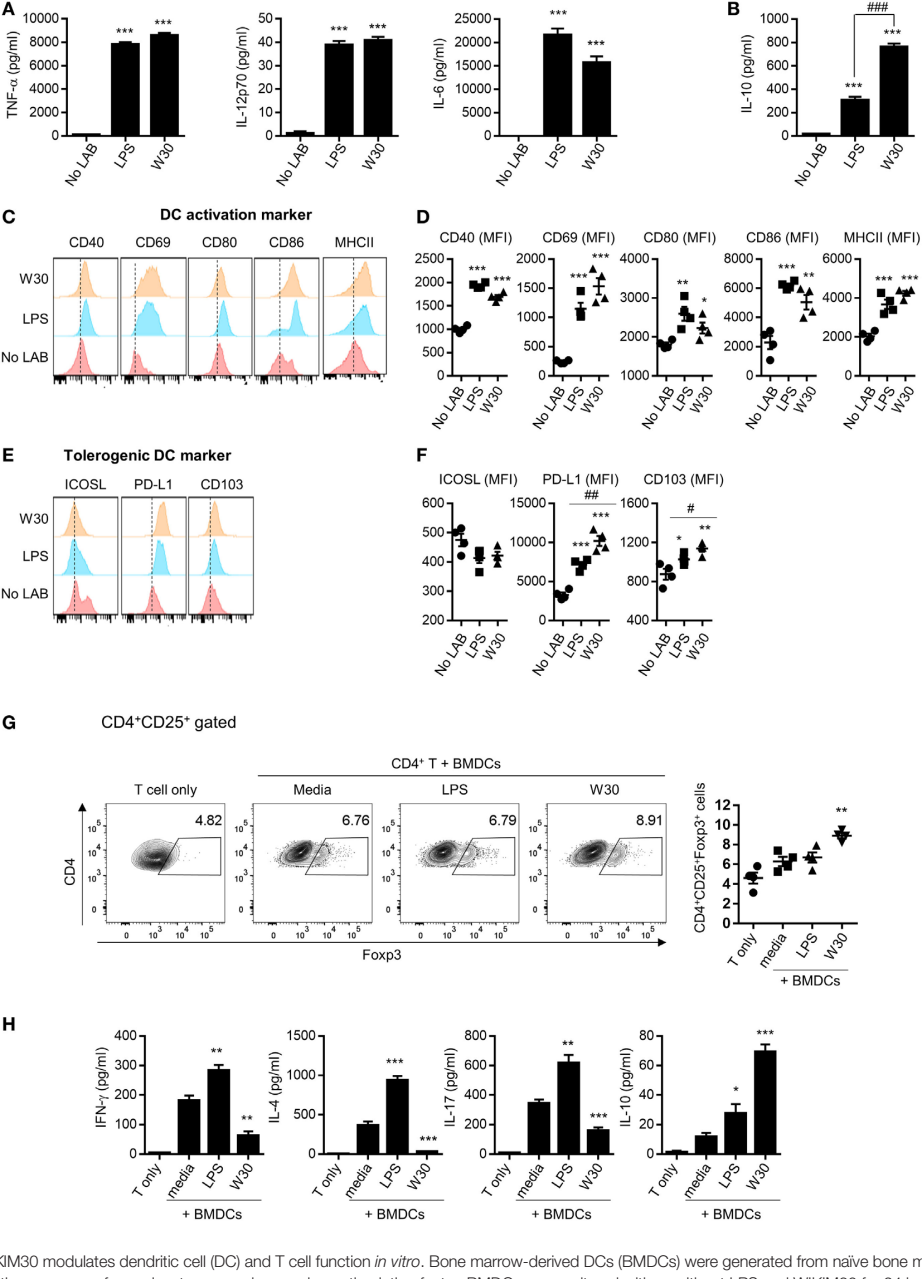
WIKIM30 modulates dendritic cell (DC) and T cell function *in vitro*. Bone marrow-derived DCs (BMDCs) were generated from naïve bone marrow cells of BALB/c mice in the presence of granulocyte-macrophage colony-stimulating factor. BMDCs were cultured with or without LPS and WIKIM30 for 24 h. **(A,B)** Expression levels of pro-inflammatory [TNF-α, interleukin (IL)-12p70, and IL-6] or anti-inflammatory (IL-10) cytokines in the culture supernatant measured with the cytometry bead array. **(C,D)** Surface expression of markers related to DC maturation (CD40, CD69, CD80, CD86, and MHCII) or tolerogenic DCs [inducible T-cell costimulator ligand (ICOS-L), programmed death ligand (PD-L)1, and CD103] was detected by flow cytometry. *p < 0.05 vs. no lactic acid bacteria (LAB) group; ^#^*p* < 0.05 vs. LPS group. **(E,F)** BMDCs were stimulated with LPS or WIKIM30 for 24 h and cocultured with CD4^+^ T cells isolated from BALB/c mice in the presence of anti-CD3 monoclonal antibody for 4 days, and the proportion of CD4^+^CD25^+^Foxp3^+^ regulatory T cells analyzed by flow cytometry **(G)** while expression levels of cytokines (IFN-γ, IL-4, IL-17, and IL-10) in the culture supernatant were measured with the cytometry bead array **(H)**. Data are representative of three independent experiments. Student’s *t*-test (unpaired); *p < 0.05, **p < 0.01, ***p < 0.001 vs. medium group; ^#^*p* < 0.05, ^##^*p* < 0.01, ^###^*p* < 0.001 vs. LPS group.

Th1 and Th2 immunity and immune tolerance are regulated by DCs (10). The ability of DCs to prime the T cell response depends on the expression of co-stimulatory molecules and secretion of cytokines (25). To evaluate the immunomodulatory role of WIKIM30, we examined changes in the phenotype and function of murine BMDCs following treatment with WIKIM30. We found that WIKIM30 treatment led to increased levels of inflammatory cytokines (TNF-α, IL-6, and IL-12p70) as well as immunoregulatory cytokine (IL-10) compared to unstimulated BMDCs. TNF-α, IL-6, and IL-12p70 production in WIKIM30- and LPS-treated BMDCs was comparable (Figure 1A), while IL-10 production in WIKIM30-treated BMDCs was markedly higher than that in LPS-treated BMDCs (Figure 1B). In addition, we compared the pattern of cytokines secretion by WIKIM30 treatment with that of different toll-like receptors (TLR) ligands (Figure S1 in Supplementary Material). Treatment of BMDCs with Pam3Cys-Ser-Ly4 (PAM, TLR1/2), LPS (TLR4), and Pam2CGDPKHPKSF (FSL-1, TLR6/2) induced comparable levels of TNF-α, and IL-6, whereas only LPS induced that of IL-12p70 with WIKIM30. IL-10 production was observed in LPS- and FSL-1-treated BMDCs, which were lower than that in WIKIM30-treated BMDCs. Next, we analyzed the expression of surface molecules including markers of activated DCs (CD40, CD69, CD80, CD86, and MHCII) and tolerogenic DCs (ICOS-L, PD-L1, and CD103) in BMDCs treated with WIKIM30 and found that CD40, CD69, CD80, CD86, and MHCII levels were comparable to those in LPS-treated BMDCs (Figures 1C,D). However, tolerogenic DC markers such as PD-L1 and CD103 were more highly expressed in WIKIM30-treated than in LPS-treated BMDCs (Figures 1E,F).

Since tolerogenic DCs are known to have an immunoregulatory effect on CD4^+^ T cells (26, 27), we investigated whether WIKIM30-treated BMDCs promote the differentiation of naïve T cells into Tregs. The generation of CD4^+^CD25^+^Foxp3^+^ Tregs was significantly increased in CD4^+^ T cells cocultured with WIKIM30-treated BMDCs compared to those cultured with untreated BMDCs (Figure 1G). Indeed, WIKIM30 treatment increased the levels of IL-10 but not of IL-4, IL-17, or IFN-γ in the supernatant (Figure 1H). These results indicate that WIKIM30 can induce the differentiation of tolerogenic DCs, which in turn promote the generation of CD4^+^CD25^+^Foxp3^+^ T cells.

### Oral Administration of WIKIM30 Suppresses DNCB-Induced AD

**Figure 2:**
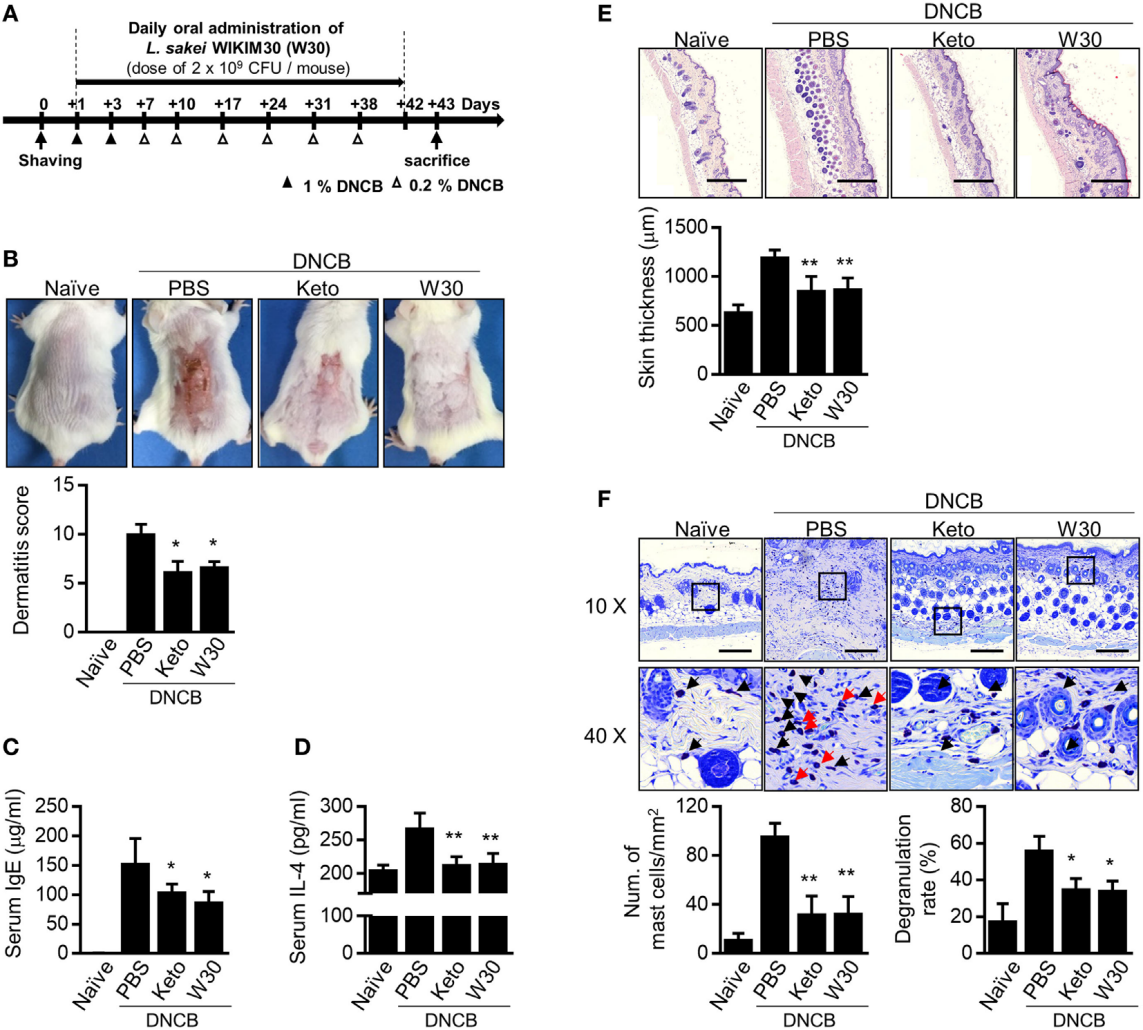
Oral administration of *Lactobacillus sakei* WIKIM30 ameliorates atopic dermatitis (AD)-like symptoms. **(A)** Experimental design. To induce AD-like immunologic features and skin lesions in mice, DNCB was applied to the dorsal skin. Phosphate-buffered saline (PBS), ketotifen, or *L. sakei* WIKIM30 was orally administered once daily for 6 weeks. **(B)** AD-like skin lesions were evaluated by visual observation. **(C,D)** Serum immunoglobulin (Ig)E and interleukin (IL)-4 levels were detected by sandwich enzyme-linked immunosorbent assay. **(E,F)** Dorsal skin sections were stained with hematoxylin and eosin or Toluidine Blue. Skin thickness and cell infiltration **(E)** and mast cell number and degranulation **(F)** were evaluated by histological analysis. Data represent the mean ± SE of *n* = 5 mice per group in three independent experiments. Student’s *t*-test (unpaired); *p < 0.05, **p < 0.01 vs. AD group.

To clarify whether WIKIM30 modulates T cell immune responses, we measured the levels of cytokines produced by splenocytes from OVA-sensitized BALB/c mice, which were re-stimulated with OVA in the presence and absence of WIKIM30 (Figure S2 in Supplementary Material). WIKIM30 strongly inhibited the release of the Th2 cytokine IL-4, while enhancing that of IL-10 in an antigen-specific manner. Based on our observation that WIKIM30 modulates the Th1/Th2 response and induces an anti-inflammatory response *in vitro*, we investigated whether WIKIM30 can alleviate AD—which is characterized by Th2 polarization (28)—using a DNCB-induced AD mouse model. The mice were orally administered PBS (AD group), the anti-histamine agent ketotifen (Keto group), or WIKIM30 (WIKIM30 group) for 42 days (Figure 2A). WIKIM30 intake ameliorated AD-like lesions compared to the AD group. Indeed, dermatitis scores—which integrate individual scores for four dermatitis symptoms (erythema, edema, erosion, and dryness)—were significantly reduced in the WIKIM30 as compared to the AD group (Figure 2B). We next examined the effect of WIKIM30 intake on the production of IgE and IL-4, which is a hallmark of AD caused by a strong Th2 immune response. As shown in Figures 2C,D, serum IgE and IL-4 levels were markedly lower in the WIKIM30 group (39 and 19%, respectively) than in the AD group. Histological analysis showed that dorsal skin thickening and inflammatory cell infiltration in AD mice were reversed, as determined by H&E staining (Figure 2E), whereas the accumulation and degranulation of mast cells, which mediate hypersensitivity by releasing inflammatory mediators, was suppressed by WIKIM30 treatment relative to the AD group, as determined by Toluidine Blue staining (Figure 2F). Collectively, these data demonstrate that oral administration of WIKIM30 effectively alleviates AD-like symptoms.

### WIKIM30 Suppresses Th2 Immune Responses in PLNs

**Figure 3:**
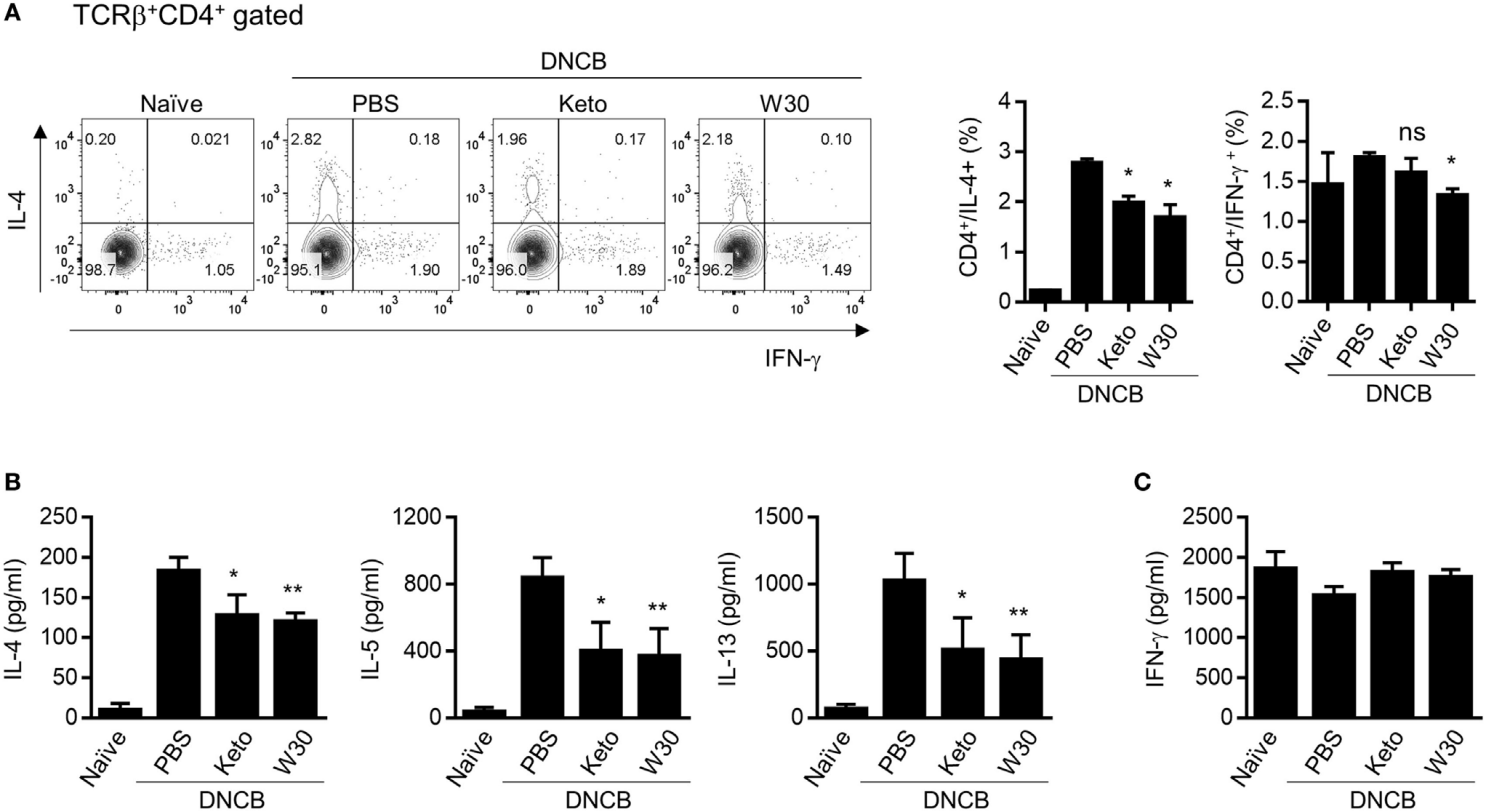
Inhibitory effect of T helper 2 immune responses in PLN cells of atopic dermatitis (AD) mice treated with WIKIM30. **(A)** PLN cells isolated from each group were treated with PMA/ionomycin/brefeldin A for 4 h and stained for intracellular cytokines [interleukin (IL)-4 and IFN-γ]. The percentages of IFN-γ^+^CD4^+^ and IL-4^+^CD4^+^ T cells were measured by flow cytometry. **(B,C)** PLN cells were treated with anti-CD3/CD28 monoclonal antibodies for 48 h, and IL-4, IL-5, and IL-13 and IFN-γ levels in the culture supernatant were measured with a cytometric bead array kit. Data represent the mean ± SE of *n* = 5 mice per group from three independent experiments. Student’s *t*-test (unpaired); *p < 0.05, **p < 0.01 vs. AD group.

The inguinal and axillary LNs located near AD-like skin lesions are active sites of immune cell accumulation, proliferation, and differentiation. Compared to naïve mice, total numbers of lymphocytes including CD4^+^ T cells, CD8^+^ T cells, and CD19^+^ B cells were expanded in PLNs with AD, but WIKIM30 treatment significantly inhibited lymphocyte recruitment to PLNs (Figure S3 in Supplementary Material). To determine whether oral administration of WIKIM30 regulates the Th1/Th2 immune balance in lymphoid organs, we examined the frequencies of CD4^+^ cells expressing IL-4 and IFN-γ at a single-cell level by intracellular cytokine staining. There were more IL-4-producing CD4^+^ T cells in AD mice, while the frequency of IL-4-producing CD4^+^ T cells was lower in the WIKIM30 group than in AD group mice. Meanwhile, the frequency of CD4^+^ T cells expressing IFN-γ was comparable across groups (Figure 3A). We also measured the levels of Th1 and Th2 cytokines secreted in the supernatant following polyclonal stimulation of PLN cells. WIKIM30 treatment reduced the levels of IL-4, IL-5, and IL-13 relative to the AD group (Figure 3B). However, IFN-γ production was comparable across groups (Figure 3C). These results indicate that WIKIM30 exerts a protective effect against AD by suppressing the Th2 immune response.

### WIKIM30 Promotes Treg Differentiation and Function in MLNs

**Figure 4:**
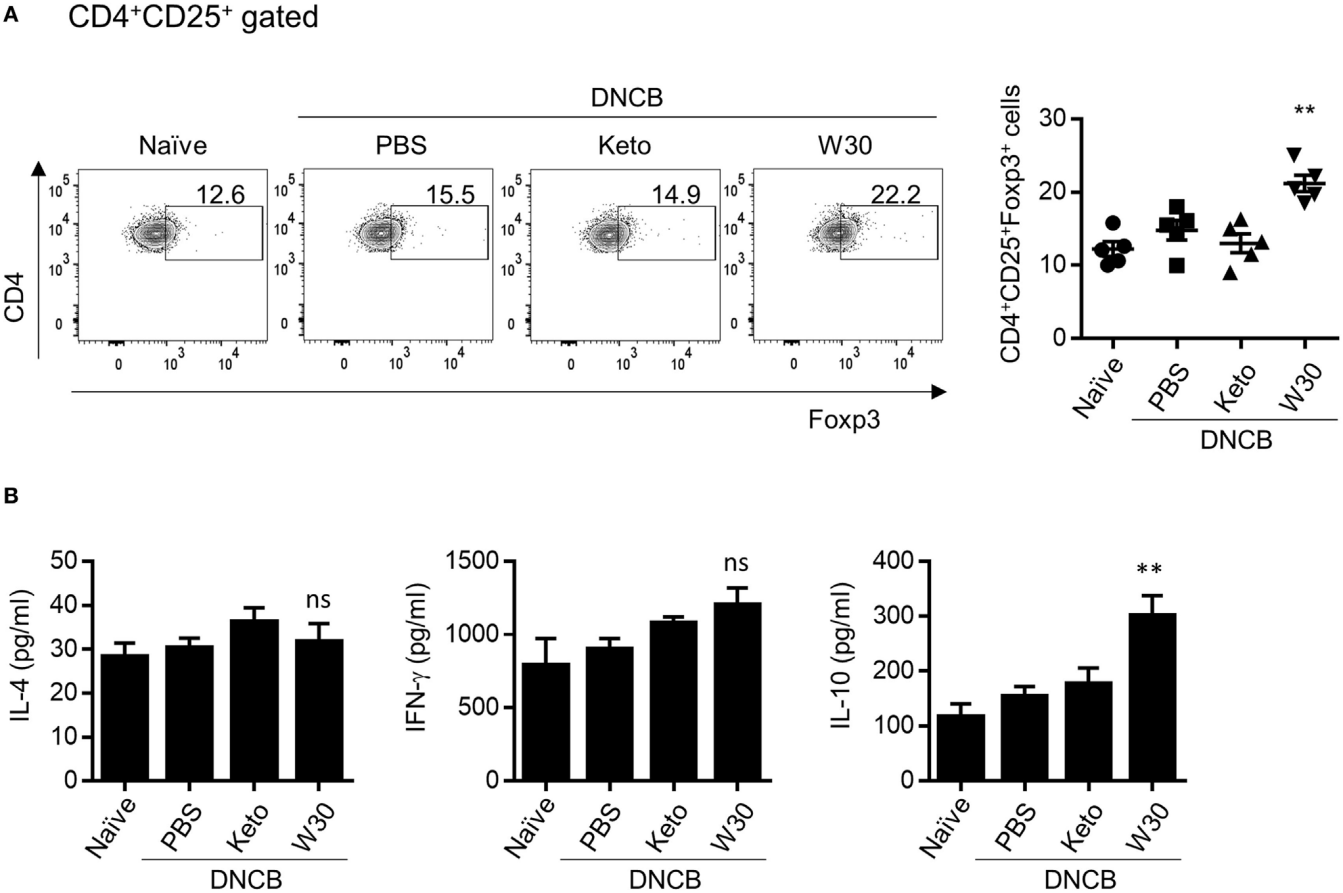
Modulatory effect of WIKIM30 on regulatory T cell (Treg)-related responses in mesenteric lymph nodes (MLNs) of atopic dermatitis (AD) mice. **(A)** Proportion of Tregs in MLNs. Isolated MLN cells were stained with antibodies against CD3, CD4, CD25, and Foxp3 and then analyzed by flow cytometry. **(B)** MLN cells were treated with anti-CD3/CD28 monoclonal antibodies for 48 h, and interleukin (IL)-4, IL-10, and IFN-γ levels in the culture supernatant were measured with the cytometric bead array kit. Data represent the mean ± SE of *n* = 5 mice per group from three independent experiments. Student’s *t*-test (unpaired); **p < 0.01 vs. AD group. ns, *p* > 0.05 vs. AD group.

The gut mucosal immune system contributes to immune tolerance by modulating the ratio of effector T cells to Tregs. We therefore investigated whether WIKIM30 intake influences immunomodulation in the gut-draining lymph nodes. WIKIM30 treatment significantly increased the MLN CD4^+^CD25^+^Foxp3^+^ Treg population compared to all other groups (Figure 4A). IL-10 production in polyclonally stimulated MLN cells showed a similar trend, while IL-4 and IFN-γ levels were comparable across groups (Figure 4B). The movement of Tregs into inflammatory draining lymph nodes is critical for the acquisition of immunosuppression (15, 16). The CD4^+^CD25^+^Foxp3^+^ Treg population in PLNs was also increased by WIKIM30 treatment (Figure S4 in Supplementary Material). These results suggest that oral administration of WIKIM30 induces Treg differentiation and IL-10 production, thereby suppressing the Th2-dominant immune response in AD.

### WIKIM30 Modulates Gut Microbiota Composition in AD Mice

**Figure 5:**
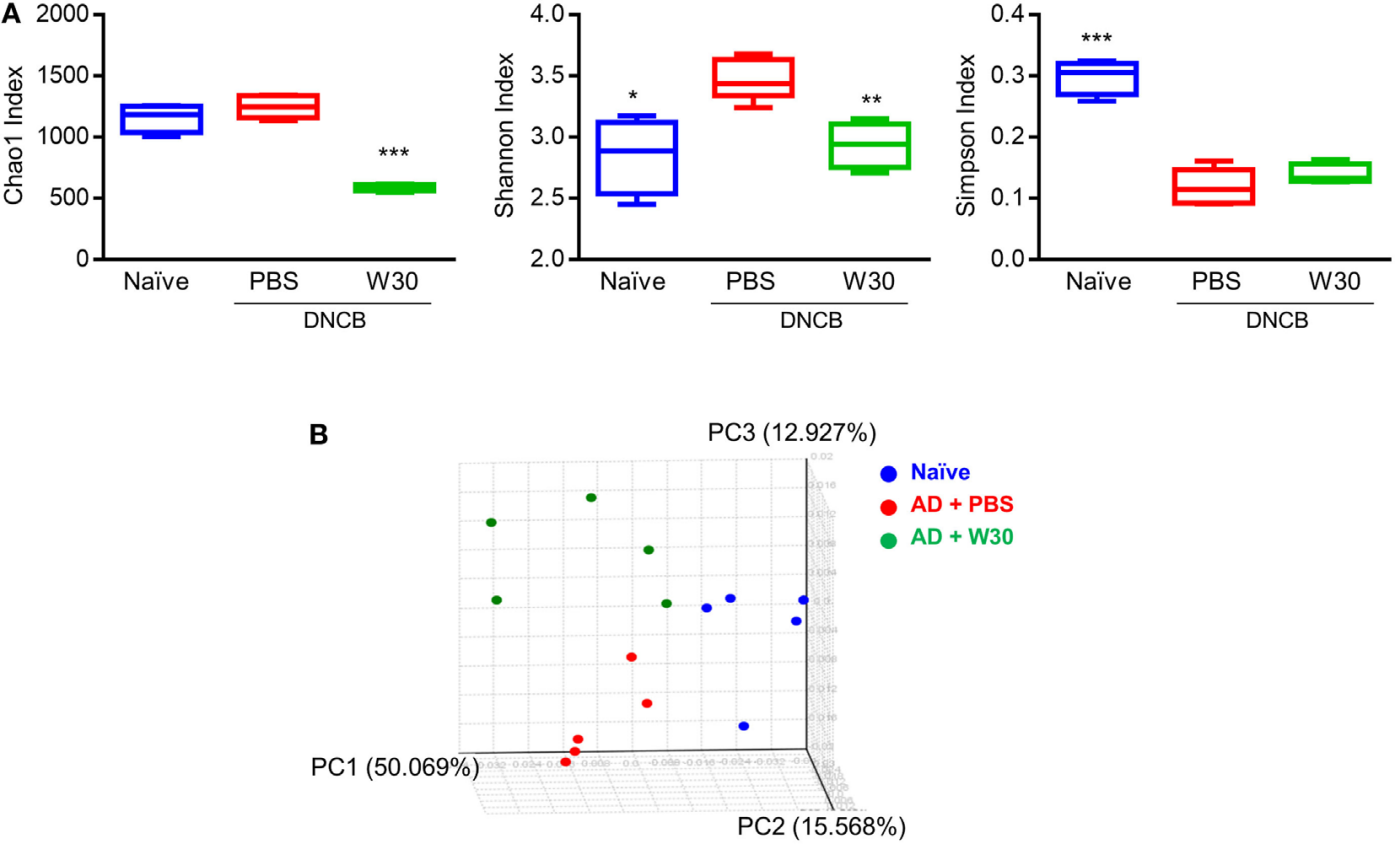
Effect of WIKIM30 on alpha and beta diversity. Alpha and beta diversity of gut microbiota in normal mice and in atopic dermatitis (AD) mice without or with WIKIM30 treatment was evaluated. **(A)** Richness of gut microbiota is indicated by the Chao1 index; diversity is indicated by the Shannon and Simpson indices. Student’s *t*-test (unpaired); *p < 0.05, **p < 0.01, ***p < 0.001 vs. AD group. **(B)** Beta diversity pattern determined by principal coordinate analysis. Naïve, normal mice (blue); AD, DNCB-induced AD mice (red); W30, DNCB-induced AD mice with oral administration of WIKIM30 (green).

Emerging evidence has shown that dysbiosis of gut microbiota is associated with AD development. To determine whether WIKIM30 can counter this phenomenon, we analyzed fecal bacteria obtained from mice in each group by 16S rRNA high-throughput amplicon sequencing. After quality filtering, 1,491,125 high-quality sequences remained, with an average of 99,408 reads per sample (range: 53,518–144,901). The Wilcoxon rank-sum test was used to determine the richness and diversity indices of each group (Figure 5A). The Chao1 index indicated that the richness of fecal microbiota was lower in the WIKIM30 group than in the naïve and AD groups. According to the Shannon and Simpson diversity index, fecal microbiota diversity was increased in AD as compared to the naïve group, but this was restored by WIKIM30 treatment (Figure 5A). A beta diversity analysis using the Bray–Curtis similarity index and principal coordinate analysis plots generated from the calculated beta diversity revealed differences in gut microbiota composition among the three groups (Figure 5B).

**Figure 6:**
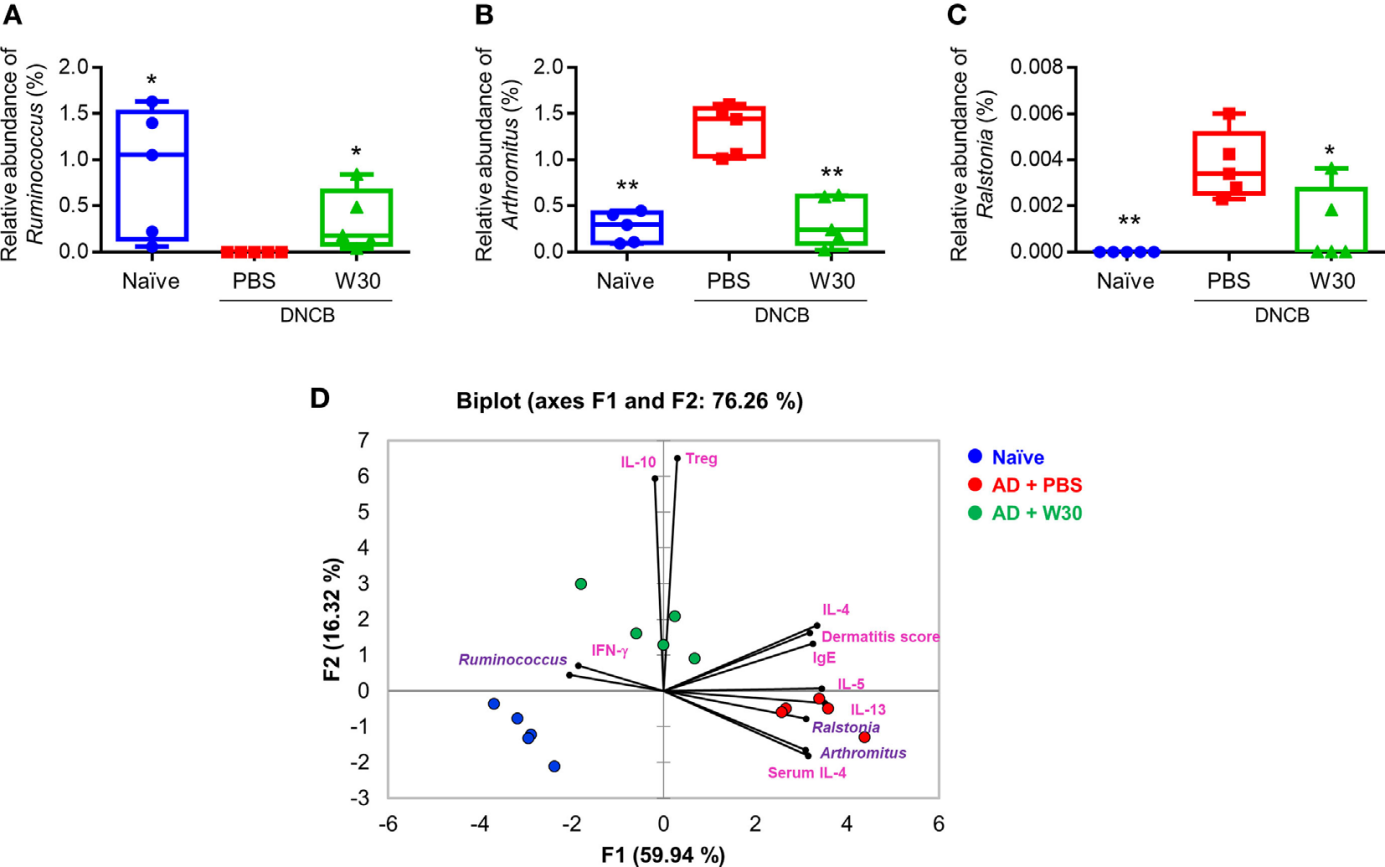
Effect of WIKIM30 on gut microbiota composition. Composition of the gut microbiota in normal mice and in atopic dermatitis (AD) mice without or with WIKIM30 treatment. **(A–C)** Relative abundance of *Ruminococcus, Arthromitus*, and *Ralstonia* in each group. Student’s *t*-test (unpaired); *p < 0.05, **p < 0.01 vs. AD group. **(D)** Correspondence analysis of fecal microbiome composition and immune response. Naïve, normal mice (blue); AD, DNCB-induced AD mice (red); W30, DNCB-induced AD mice with oral administration of WIKIM30 (green).

To determine whether the protective effects of WIKIM30 against AD are correlated with alterations in the gut microbiota, we analyzed differences in bacterial abundance among groups. To identify the specific bacterial taxa in each group, gut microbiota compositions were compared by the linear discriminant analysis effect size method (Figure S5 in Supplementary Material). At the genus level, the AD-associated decrease in the relative abundance of *Ruminococcus* was restored by WIKIM30 treatment (Figure 6A). Indeed, the relative abundance of *Arthromitus* and *Ralstonia* increased by AD sensitization was reversed by WIKIM30 treatment (Figures 6B,C). We also performed a correspondence analysis of fecal microbiota composition to determine the effect of WIKIM30 on immune response (Figure 6D; Figure S6 in Supplementary Material). Alterations in the abundance of genus *Arthromitus* and *Ralstonia* were positively correlated with Th2-related responses in AD, whereas the change in *Ruminococcus* abundance was positively correlated with Treg-related responses induced by WIKIM30 treatment. These data indicate that WIKIM30 may restore immune balance in AD through modulation of the gut microbiota.

## Discussion

In this study, we investigated whether *L. sakei* WIKIM30—a probiotic strain isolated from kimchi—can alleviate AD as well as the underlying mechanism through *in vitro* and *in vivo* studies. Initiation of an immune response by ingested probiotics depends on antigen-presenting cells in the GI tract, including DCs (21). DCs recognize bacterial components (e.g., pathogen-associated molecular patterns) through pattern recognition receptors (PRRs) such as TLRs (29). Thus, DCs stimulated by specific bacterial components on TLRs or pathogen-derived molecules can promote different immune responses by inducing distinct T cell subtypes. For example, LPS from *Escherichia coli* (30), flagellin (31), viral double-stranded RNA (32), and bacterial CpG DNA (33) promote the Th1 response, whereas LPS from *Porphyromonas gingivalis* (34), helminth components (35), and cholera toxin (36) promote the Th2 response. Filamentous hemagglutinin from *Bordetella pertussis* stimulated DCs that enhanced Treg differentiation and showed increased IL-10 production (37). Likewise, different bacteria including WIKIM30 variably influence DC function, which can be detected based on the expression patterns of cytokines and surface molecules. Treatment of BMDCs with WIKIM30 stimulated the production of both pro- and anti-inflammatory cytokines and increased the activation of tolerogenic DCs, suggesting that WIKIM30 contains factors that modulate immune activation and tolerance (Figures 1A,B; Figure S1 in Supplementary Material). Collectively, these data indicate that WIKIM30 may contain immune modulatory components that engage PRRs like TLR1/2, TLR4, and TLR6/2; identifying these can provide insight into the mechanism of DC polarization toward a tolerogenic phenotype. Moreover, our coculture experiments using WIKIM30-treated BMDCs and naïve CD4^+^ T cells showed that WIKIM30-treated DCs increased the Treg-related response while decreasing responses related to Th1, Th2, and Th17 (Figure 1F), providing evidence that WIKIM30-treated BMDCs are polarized toward tolerogenic DCs.

To investigate the immune modulatory effects of WIKIM30 *in vivo*, we established a mouse model of DNCB-induced AD, which exhibits a polarized Th2 response. Oral administration of WIKIM30 inhibited the production of serum IgE and IL-4—a Th2-associated immune response—in these mice (Figure 2). WIKIM30 treatment restored the skin epidermis and decreased the number of CD4^+^ T cells and B cells as well as Th2 cytokine expression in the PLN (Figure 3) of AD model mice, whereas the proportion of Tregs in the MLN (Figure 4) as well as in the PLN (Figure S4) was increased in the WIKIM30-treated group. Tregs can rapidly migrate into inflamed draining lymph nodes and tissue by expressing chemokine receptors [C-C chemokine receptor type (CCR)4 and CCR6] (16). These data demonstrate that the movement of Tregs generated by WIKIM30 administration in MLN into inflammatory sites may inhibit the Th2-dominant immune response of AD; thus, WIKIM30 may be a tolerogenic DC inducer in the GI tract. However, additional studies are needed to confirm the direct action of WIKIM30 in promoting tolerogenic DC generation in the GI immune system.

Previous studies have reported that changes in gut microbiota profiles contribute to the development of AD by affecting the Th2-dominant immune response. In human studies, infants with AD showed increased populations of *Faecalibacterium prausnitzii* subspecies (38) and *Staphylococcus aureus* (39). Furthermore, the abundance of genus *Clostridium* was increased whereas that of *Bacteroides* was decreased in AD patients (40). Although we were unable to detect *F. prausnitzii* or *S. aureus* in AD mice, the abundance of *Clostridium* and *Bacteroides* showed similar patterns to those in humans. Among the mice with oxazolon-induced AD, mice with higher susceptibility to AD showed increased abundance of *Bacteroides uniformis* as well as species belonging to the family Lachnospiraceae and an unclassified genus of the family Rikenellaceae, whereas those with lower susceptibility showed greater numbers of unclassified *Bacteroides* species (41).

In this study, we found that oral administration of WIKIM30 modulates the structure of gut microbiota that may influence allergic immune responses in AD mice. The proportion of genus *Ruminococcus* was significantly decreased in AD, but this was restored by WIKIM30 treatment; thus, an increase in the relative abundance of *Ruminococcus* may be positively correlated with Treg-related responses induced by WIKIM30. On the contrary, the relative abundance of *Arthromitus* and *Ralstonia* were elevated in AD and were reduced by WIKIM30 treatment, indicating that increases in the abundance of these two genera may be positively correlated with Th2-related responses in AD (Figure 6D). Previous studies have described the roles of *Ruminococcus, Arthromitus*, and *Ralstonia* in immune responses. For instance, *Ruminococcus albus, R. bromii*, and *R. callidus* are more highly represented in healthy individuals than in Crohn’s disease patients (42), whereas *Ruminococcus* numbers are reduced in psoriatic arthritis patients (43). *Arthromitus* is a commensal segmented filamentous bacterial species that is known to induce the differentiation of CD4^+^ T cells into Th17 cells (44). In addition, *Ralstonia* is more abundant in patients with asthma than in those with non-asthmatic chronic rhinosinusitis (45) and is associated with the inflammatory response in Parkinson’s disease (46) and the mucosal Th2 response in food allergy (47). These data demonstrate that WIKIM30 alleviates AD symptoms by increasing Treg-related or decreasing AD-related gut bacteria. However, mechanism on how probiotics directly alter gut microbiota structure remains to be determined.

In conclusion, our results showed that WIKIM30 stimulates the generation of Tregs directly by inducing tolerogenic DCs or indirectly by modulating gut microbiome profiles to suppress immune responses in AD. These findings suggest that administration of WIKIM30 modulates the gut microbiome, which can be an effective therapeutic strategy for alleviating AD.

## Ethics Statement

All animal procedures were performed according to the National Institutes of Health Guidelines for the Humane Treatment of Animals with approval from the Institutional Animal Care and Use Committee of the World Institutes of Kimchi (WIKIM IACUC 201509, 201601, and 201701).

## Author Contributions

H-JC, M-SK, and SKL conceived and designed the experiments; M-SK, SKL, J-YJ, JL, HKP, NK, MY, M-YS, and HEJ performed the experiments; M-SK, SKL, J-YJ, YJO, SWR, and H-JC analyzed the data; M-SK, SKL, and H-JC prepared the manuscript; and H-JC supervised the study.

## Conflict of Interest Statement

The authors declare that the research was conducted in the absence of any commercial or financial relationships that could be construed as a potential conflict of interest.
