# Supplementary material for: Lactobacillus sakei WIKIM30 Ameliorates Atopic Dermatitis-Like Skin Lesions by Inducing Regulatory T Cells and Altering Gut Microbiota Structure in Mice
Source: Front Immunol. 2018 Aug 14;9:1905. doi: 10.3389/fimmu.2018.01905 (PMC6102352; doi:10.3389/fimmu.2018.01905)
Supplement: Supplementary file 1 [file Presentation_1.pptx]

## Slide 1
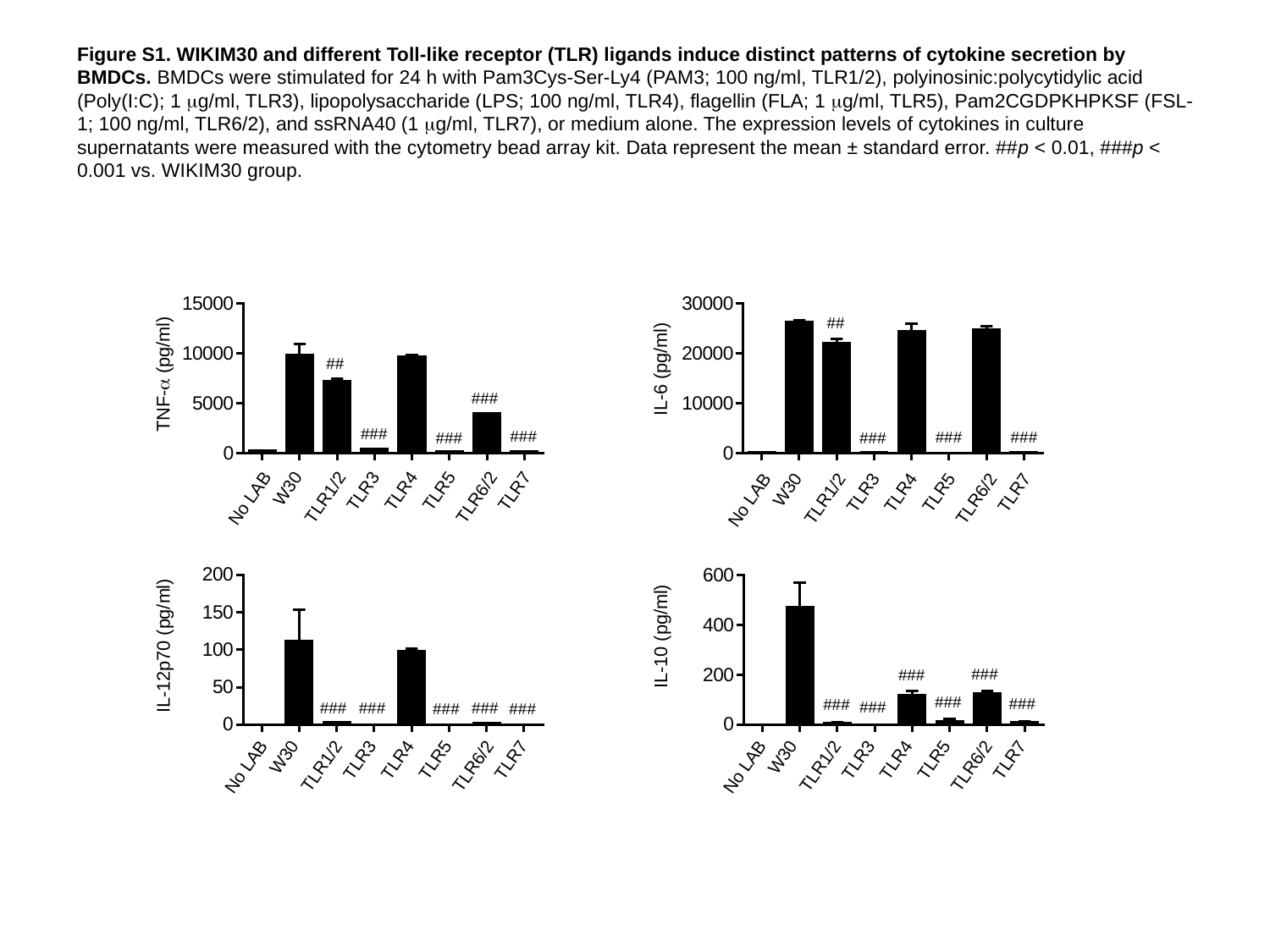

Figure S1. WIKIM30 and different Toll-like receptor (TLR) ligands induce distinct patterns of cytokine secretion by BMDCs. BMDCs were stimulated for 24 h with Pam3Cys-Ser-Ly4 (PAM3; 100 ng/ml, TLR1/2), polyinosinic:polycytidylic acid (Poly(I:C); 1 mg/ml, TLR3), lipopolysaccharide (LPS; 100 ng/ml, TLR4), flagellin (FLA; 1 mg/ml, TLR5), Pam2CGDPKHPKSF (FSL-1; 100 ng/ml, TLR6/2), and ssRNA40 (1 mg/ml, TLR7), or medium alone. The expression levels of cytokines in culture supernatants were measured with the cytometry bead array kit. Data represent the mean ± standard error. ##p < 0.01, ###p < 0.001 vs. WIKIM30 group.
##
##
IL-6 (pg/ml)
TNF-a (pg/ml)
###
###
###
###
###
###
###
W30
TLR3
TLR4
TLR5
TLR7
TLR1/2
TLR6/2
No LAB
W30
TLR3
TLR4
TLR5
TLR7
TLR1/2
TLR6/2
No LAB
IL-10 (pg/ml)
IL-12p70 (pg/ml)
###
###
###
###
###
###
###
###
###
###
###
W30
TLR3
TLR4
TLR5
TLR7
TLR1/2
TLR6/2
No LAB
W30
TLR3
TLR4
TLR5
TLR7
TLR1/2
TLR6/2
No LAB

## Slide 2
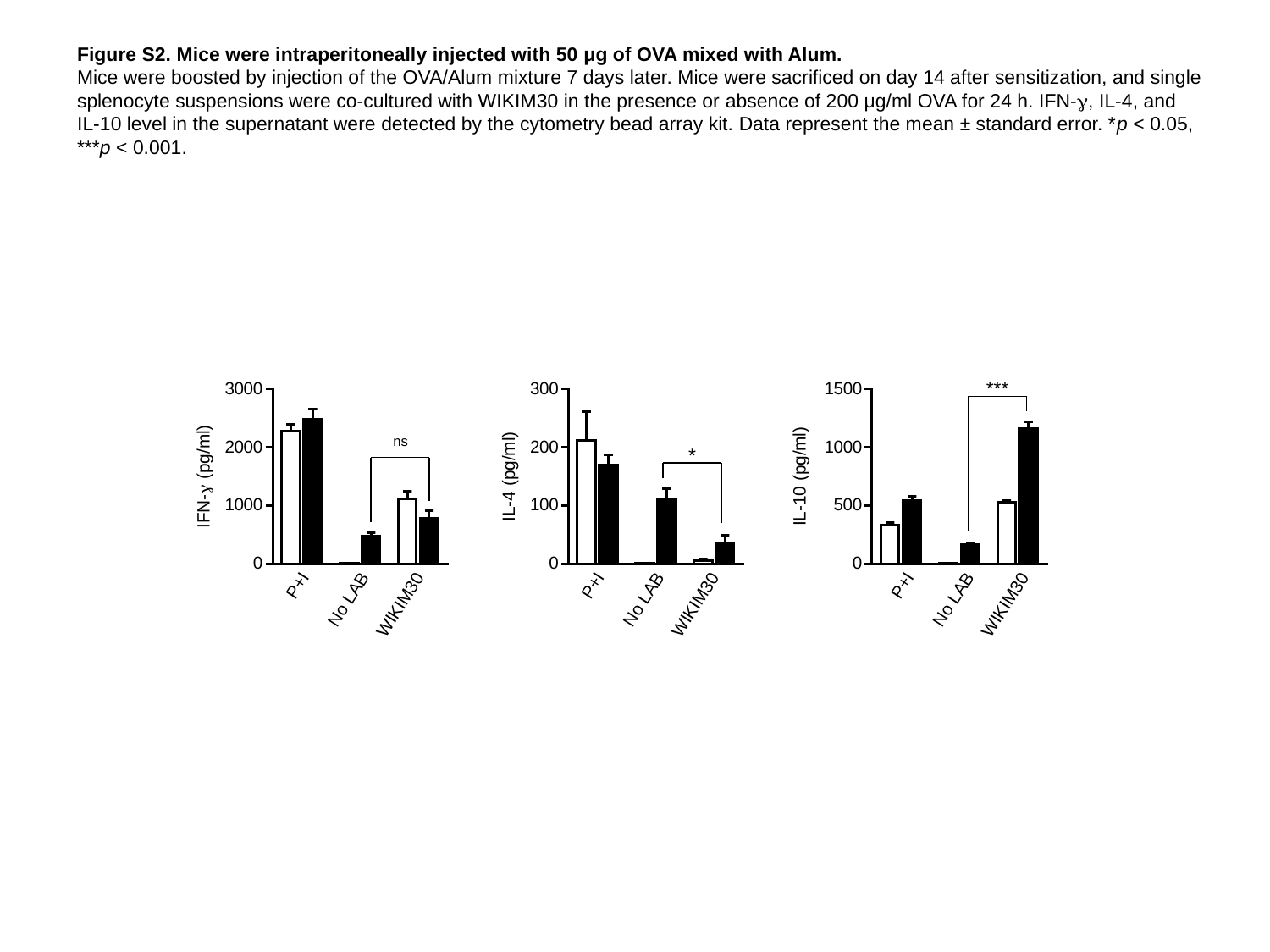

Figure S2. Mice were intraperitoneally injected with 50 μg of OVA mixed with Alum.
Mice were boosted by injection of the OVA/Alum mixture 7 days later. Mice were sacrificed on day 14 after sensitization, and single splenocyte suspensions were co-cultured with WIKIM30 in the presence or absence of 200 μg/ml OVA for 24 h. IFN-g, IL-4, and IL-10 level in the supernatant were detected by the cytometry bead array kit. Data represent the mean ± standard error. *p < 0.05, ***p < 0.001.
***
ns
*
IL-4 (pg/ml)
IFN-g (pg/ml)
IL-10 (pg/ml)
P+I
No LAB
WIKIM30
P+I
No LAB
WIKIM30
P+I
No LAB
WIKIM30

## Slide 3
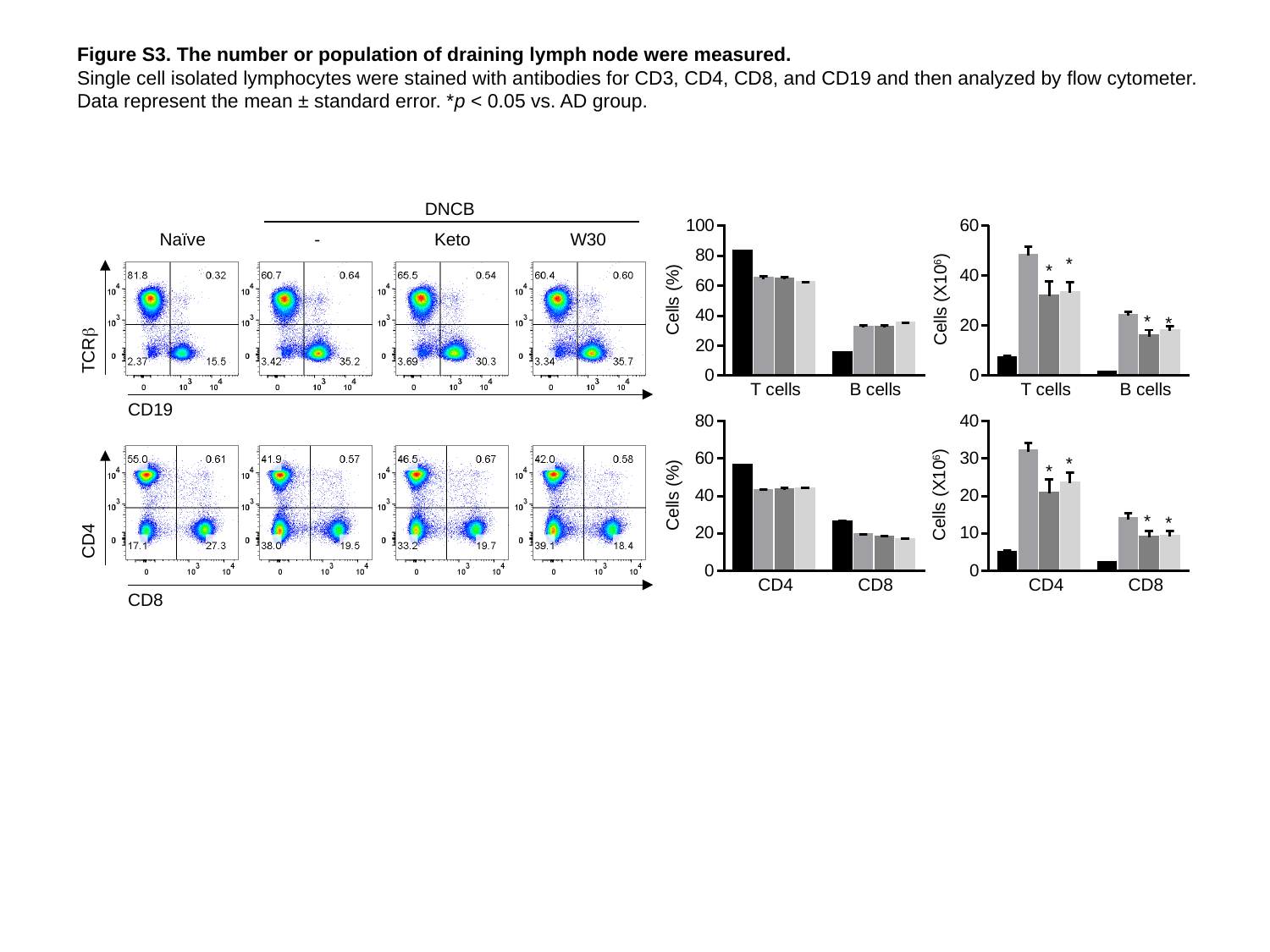

Figure S3. The number or population of draining lymph node were measured.
Single cell isolated lymphocytes were stained with antibodies for CD3, CD4, CD8, and CD19 and then analyzed by flow cytometer. Data represent the mean ± standard error. *p < 0.05 vs. AD group.
DNCB
Naïve
-
Keto
W30
*
*
Cells (%)
Cells (X106)
*
*
TCRb
B cells
B cells
T cells
T cells
CD19
*
*
Cells (%)
Cells (X106)
*
*
CD4
CD8
CD8
CD4
CD4
CD8

## Slide 4
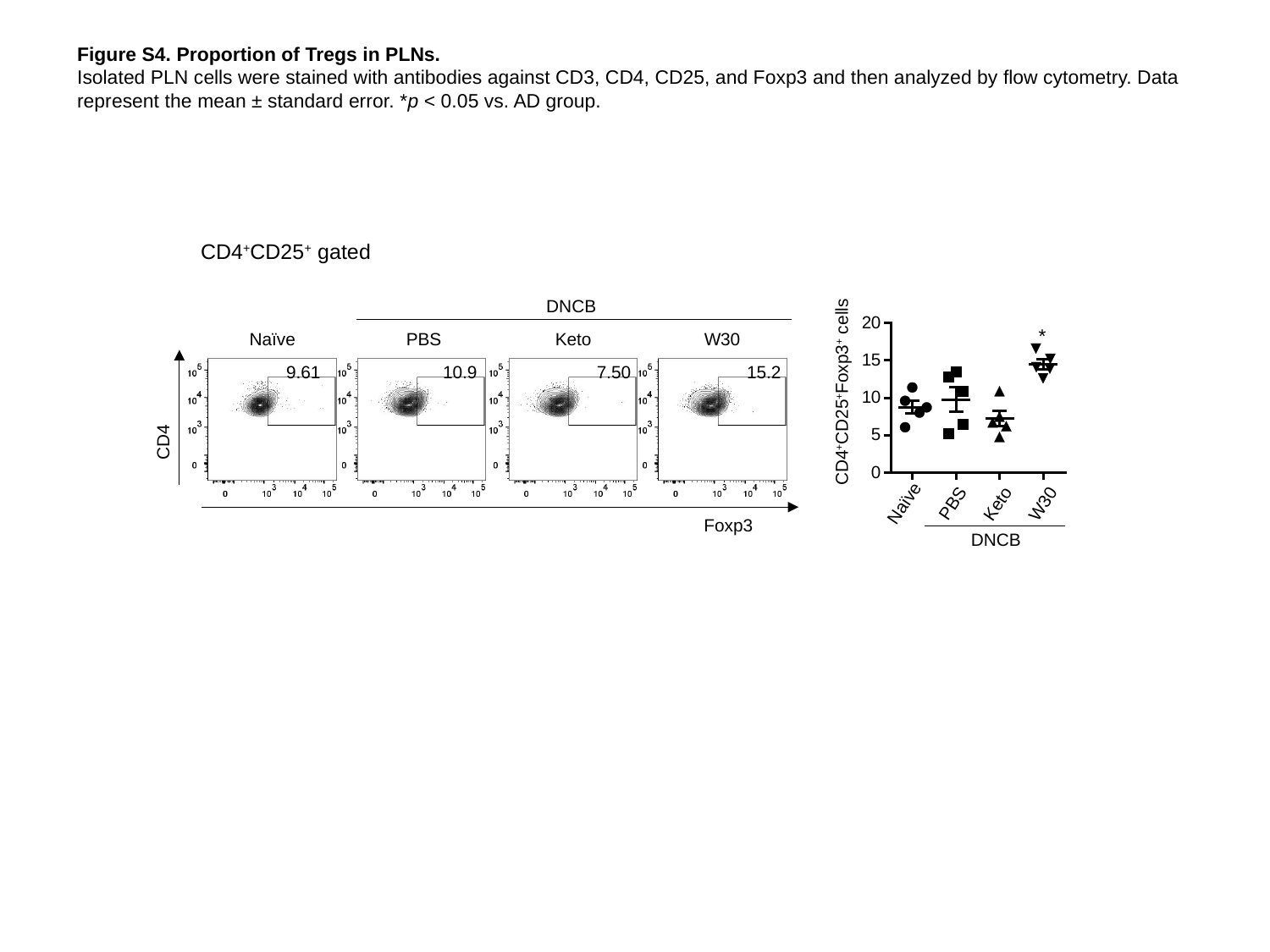

Figure S4. Proportion of Tregs in PLNs.
Isolated PLN cells were stained with antibodies against CD3, CD4, CD25, and Foxp3 and then analyzed by flow cytometry. Data represent the mean ± standard error. *p < 0.05 vs. AD group.
CD4+CD25+ gated
DNCB
*
Naïve
PBS
Keto
W30
9.61
10.9
7.50
15.2
CD4+CD25+Foxp3+ cells
CD4
Naïve
PBS
Keto
W30
DNCB
Foxp3

## Slide 5
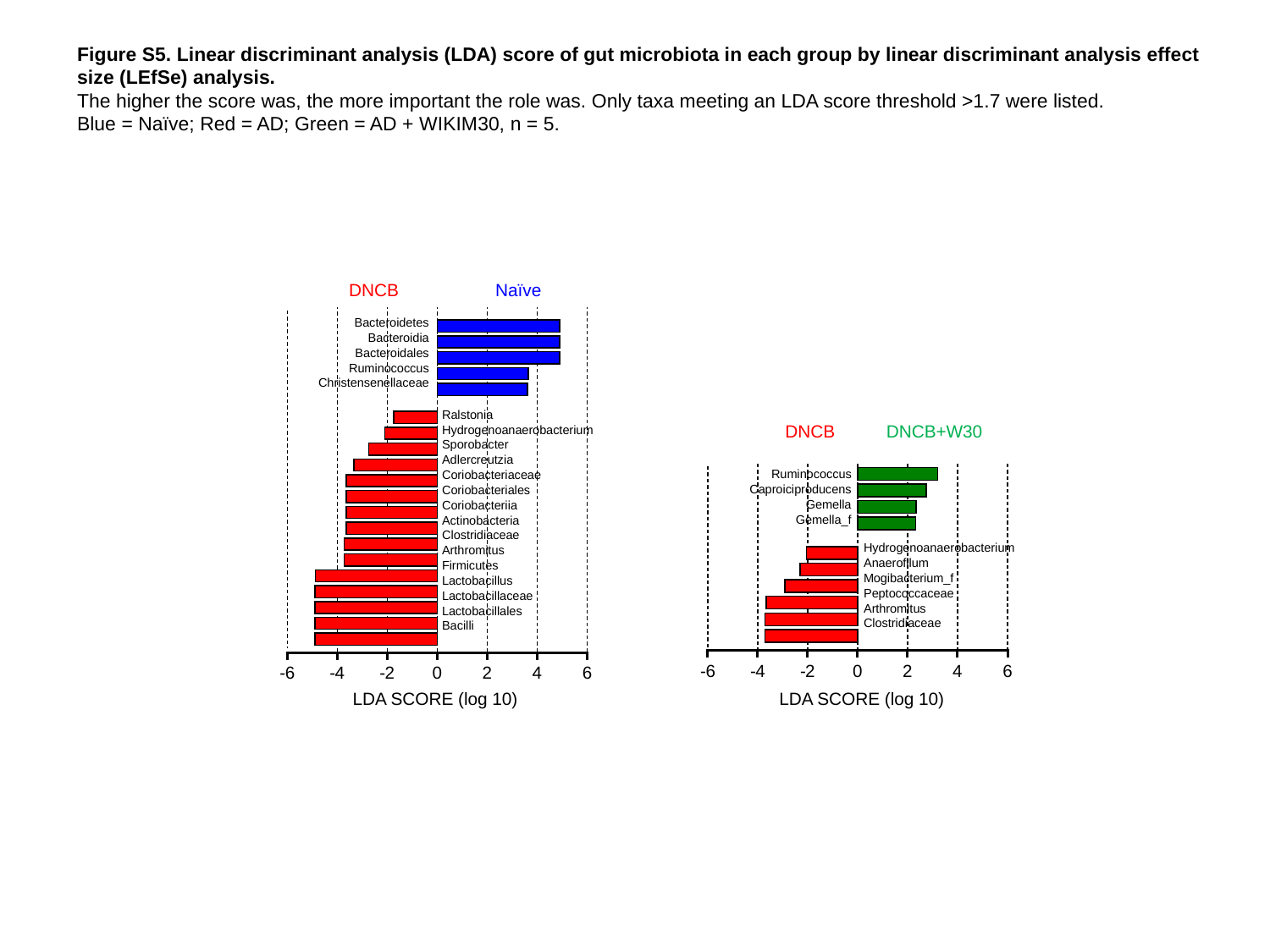

Figure S5. Linear discriminant analysis (LDA) score of gut microbiota in each group by linear discriminant analysis effect size (LEfSe) analysis.
The higher the score was, the more important the role was. Only taxa meeting an LDA score threshold >1.7 were listed.
Blue = Naïve; Red = AD; Green = AD + WIKIM30, n = 5.
DNCB
Naïve
| Bacteroidetes |
| --- |
| Bacteroidia |
| Bacteroidales |
| Ruminococcus |
| Christensenellaceae |
| Ralstonia |
| --- |
| Hydrogenoanaerobacterium |
| Sporobacter |
| Adlercreutzia |
| Coriobacteriaceae |
| Coriobacteriales |
| Coriobacteriia |
| Actinobacteria |
| Clostridiaceae |
| Arthromitus |
| Firmicutes |
| Lactobacillus |
| Lactobacillaceae |
| Lactobacillales |
| Bacilli |
DNCB
DNCB+W30
| Ruminococcus |
| --- |
| Caproiciproducens |
| Gemella |
| Gemella\_f |
| Hydrogenoanaerobacterium |
| --- |
| Anaerofilum |
| Mogibacterium\_f |
| Peptococcaceae |
| Arthromitus |
| Clostridiaceae |
| |
LDA SCORE (log 10)
LDA SCORE (log 10)

## Slide 6
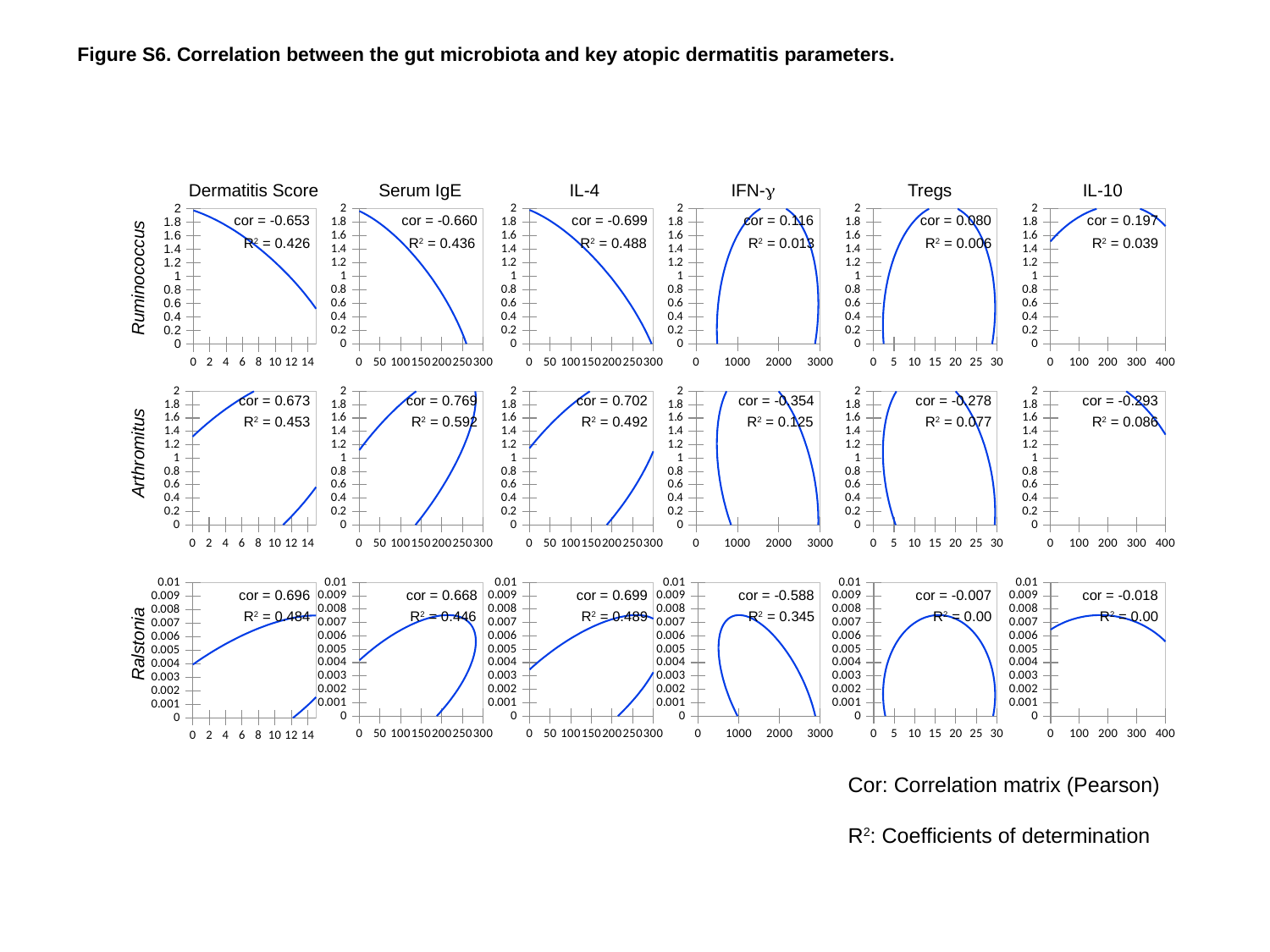

Figure S6. Correlation between the gut microbiota and key atopic dermatitis parameters.
Dermatitis Score
Serum IgE
IL-4
IFN-g
Tregs
IL-10
### Chart
| Category | | |
|---|---|---|
### Chart
| Category | | |
|---|---|---|
### Chart
| Category | | |
|---|---|---|
### Chart
| Category | | |
|---|---|---|
### Chart
| Category | | |
|---|---|---|
### Chart
| Category | | | |
|---|---|---|---|cor = -0.653
cor = -0.660
cor = -0.699
cor = 0.116
cor = 0.080
cor = 0.197
R2 = 0.426
R2 = 0.436
R2 = 0.488
R2 = 0.013
R2 = 0.006
R2 = 0.039
Ruminococcus
### Chart
| Category | | |
|---|---|---|
### Chart
| Category | | |
|---|---|---|
### Chart
| Category | | |
|---|---|---|
### Chart
| Category | | |
|---|---|---|
### Chart
| Category | | |
|---|---|---|
### Chart
| Category | | | |
|---|---|---|---|cor = 0.673
cor = 0.769
cor = 0.702
cor = -0.354
cor = -0.278
cor = -0.293
R2 = 0.453
R2 = 0.592
R2 = 0.492
R2 = 0.125
R2 = 0.077
R2 = 0.086
Arthromitus
### Chart
| Category | | |
|---|---|---|
### Chart
| Category | | |
|---|---|---|
### Chart
| Category | | |
|---|---|---|
### Chart
| Category | | |
|---|---|---|
### Chart
| Category | | |
|---|---|---|
### Chart
| Category | | | |
|---|---|---|---|cor = 0.696
cor = 0.668
cor = 0.699
cor = -0.588
cor = -0.007
cor = -0.018
R2 = 0.484
R2 = 0.446
R2 = 0.489
R2 = 0.345
R2 = 0.00
R2 = 0.00
Ralstonia
Cor: Correlation matrix (Pearson)
R2: Coefficients of determination
